# Moving from Histopathology to Molecular Tools in the Diagnosis of Molluscs Diseases of Concern under EU Legislation

**DOI:** 10.3389/fphys.2016.00538

**Published:** 2016-11-14

**Authors:** Raquel Aranguren, Antonio Figueras

**Affiliations:** Instituto de Investigaciones Marinas, Consejo Superior de Investigaciones CientíficasPontevedra, Spain

**Keywords:** diagnostic techniques, quantitative real time PCR, diseases, molluscs, pathology, molecular tools

## Abstract

One of the main factors limiting molluscs production is the presence of pathogens and diseases. Disease agent transfer via transfers of live molluscs has been a major cause of disease outbreaks and epizootics. Because of that, the European Union has adopted several decisions and directives, the last in 2006 (2006/88/EC) to control movements of marine organisms over the European countries. Once the disease is established in a determined area its eradication is a complicated task because life cycle of pathogens are not completely known and only a good and early diagnosis of the disease could be the most appropriate way to deal with it. Besides, molluscs do not have an adaptive immune response and vaccination strategies are not possible. Molluscs listed diseases under EU legislation are mainly protozoan parasites, that's why histological techniques are recognized for their diagnosis. However, molecular techniques are being increasingly used primarily as confirmatory techniques of the presence of the pathogens but also in disease monitoring programs. Research perspectives are mainly focussed in the optimization, of the already described techniques to gain in sensitivity and sensibility and in the development of new molecular biology techniques (quantitative real time PCRs), that are faster and easier to apply and that allow a positive diagnosis even in early stages of infection. However, molecular tools detect DNA sequences of the pathogen which does not imply that pathogen is viable in the cell host and the infection is established. Consequently, it needs to be validated against other techniques, such as histology or *in situ* hybridization, so that its reliability can be determined.

## Introduction

Aquaculture is a dynamic activity worldwide that over the past few decades has doubled its production. According to FAO statistics (FAO, 2014), the world aquaculture production in 2012 was 66, 63 million tons (not including aquatic algae and non-food products).

Asian countries are the main producers of molluscs being China by far the leading producer. In the European Union, the marine bivalve industry has grown to be very important for many regions contributing substantially to social and economic activity in the coastal zones. European aquaculture is focused on a limited number of species (oysters and mussels mainly) being raised at an industrial level. France, Spain, and Italy are the main bivalve producing countries. France is leading the oyster, *Crassotrea gigas*, production in Europe (80.000 tons/year). The mussels *Mytilus edulis* and *Mytilus galloprovincialis* are the bivalve species with the highest production output in Europe (491.000 tons/year). Spain is the third world producer and the top European producer with an output of nearly 300.000 tons/year.

There is a global trend in the growth of human consumption of seafood. Seafood constitutes an important and essential part of the diet of many people in the world and the need for increasing production will persist as the human population expands. In some countries, seafood is recognized as an important and healthy part of the human diet. Most of the demand for seafood is and will continue to be for finfish, but the production and harvest of molluscs, particularly bivalves, will also be an important rising demand. While the harvest of natural bivalve stocks will continue being significant, many wild stocks are probably already being harvested at or near maximum sustainable limits, and in some places some stocks may even be overharvested. Because of it, aquaculture is a good alternative to the harvest of wild stocks.

Bivalves are ideal animals for aquaculture: they are herbivores that require no additional feeding apart from the natural algae content of seawater and generally minimum husbandry. Although they have been cultured for hundreds of years, advances in culture technology in recent years have led to significantly higher production. Continued improvements in culture methodology and technology will be still required to meet increasing demand and also to make bivalve culture economically more attractive to both investors and people who wish to become shellfish farmers. An examination of the FAO marine aquaculture production data (FAO 2001–2014), shows that the number of bivalve species being cultured is increasing annually, and that more and more developing countries are becoming involved. Toward the end of the 19th century reliable techniques for culturing bivalves were developed. This allowed a rapid growth in production that was featly augmented about a century later by the development of hatchery techniques for seed production.

In agreement with the World Organization of Animal Health (OIE)^1^, during last decades, the world production of molluscs has been adversely affected by numerous diseases and due to his severe impact in the economic and socioeconomic development in many countries, some of these diseases have turned into a primary restriction for the development and the sustainability of the culture of molluscs. The transport of alive molluscs transferring infectious agents has been the principal reason of outbreaks of diseases and epizooties. Bearing this in mind, one of the very few ways to reduce the impact of such pathogens on commercially exploited bivalves is to establish effective programs to prevent the transfer of infected stocks. The risk associated with transfer of molluscs is particularly serious when they occur over long distances or overseas. However, a serious limiting factor is the lack of scientific information on even basic biology of molluscs pathogens.

Historically, infectious diseases have seriously affected the marine bivalve industry in Europe in various occasions. For example, in the early 1970's the Portuguese oyster, *Crassostrea angulata*, was dramatically affected by an irido-like virus (Marteil, 1976) leading to the almost extermination of the oysters in the European Atlantic waters. It was speculated that uncontrolled transfers of *C. gigas* introduced this irido virus to *C. angulata*, which was highly susceptible. In the late 1970s and early 1980s, mass mortalities attributed to Bonamiosis and marteiliosis caused declines in oyster production in Europe which favored the development of different management strategies to counteract the threat of both pathogens (Herrbach, 1971; Grizel et al., 1974; Alderman, 1979; Pichot et al., 1980; Bannister and Key, 1982; Van Banning, 1982; Polanco et al., 1984; Grizel, 1985; Friedman et al., 1989; Barber and Davis, 1994; Friedman and Perkins, 1994). Since 2008 massive mortality outbreaks affecting *Crassostrea gigas* has also being reported in different Member States attributed to the presence of the newly described oyster herpes virus μvar (Segarra et al., 2010) and a new *Marteilia* species, *Marteilia cochillia*, which is also causing a collapse in the cockle, *Cerastoderma edule*, fisheries in Galicia and Catalonia (NW and NE of Spain respectively; Carrasco et al., 2013; Villalba et al., 2014).

The control of farmed shellfish health is one of the key elements to maintain the competitiveness and to increase the sustainability of the European industry. Because of that, the European Union has adopted the Council Directive 2006/88/EC “on animal health requirements for aquaculture animals and products thereof and on the prevention and control of certain diseases in aquatic animals” to control movements over the European countries stablishing two categories of notifiable diseases including the most relevant molluscs diseases classifying them as exotic or non exotic diseases and making emphasis in the prevention, the control and the eradication of the aquatic animal diseases. Exotic diseases are those that are not presented in the European countries and if detected they must be eradicated (*Bonamia exitiosa, Microcytos mackini*, and *Prekinsus marinus*). Non exotic-diseases are generally confined to certain areas within the EU (*Bonamia ostreae* and *Marteilia refringens*). This Directive also leaves an open frame to include newly described pathogens as “emerging diseases” if they are consider to be a threat to the European aquaculture as it has been the case of the oyster herpes virus μvar (Commission regulation 175/2010)^2^. In Table 1 it is summarized the main molluscs species produced in Europe and the EU listed pathogens detected on its specie.

**Table 1:** **Main molluscs species produced in Europe (FAO, 2014) and detected pathogens controlled under EU directive**.

**Molluscs species**	**European production (Tns)**	**EU listed pathogens**	
		**Non exotic diseases**	**Exotic diseases**
*Mytilus* spp.	495.974	*Marteilia refringens*	
*Crassostrea gigas*	89.629	Herpes virus μvar^*^	*Perkinsus marinus, Microcytos mackini*
*Ruditapes phillipinarum*	31.651	Herpesvirus^**^	
*Ruditapes decussatus*	4.002	Herpesvirus^**^	
*Ostrea edulis*	2.801	*Bonamia ostreae, M. refringens*	*B. exitiosa, M. mackini*

* Emerging diseases (Commission regulation 175/2010);

** *Non listed disease*.

The effectiveness of any treatment against diseases in molluscs is limited by the physiology of the animals as well as by the characteristics of the culture system (Berthe, 2008). Molluscs are usually reared in the open sea which strongly limits the potential use of chemotherapy, because of the quantity of product to be used, its impact on the environment and the obvious risk of re-infection. Moreover, molluscs lack and adaptive immune response for what they cannot produce antibodies (Bayne, 2003; Ottaviani, 2011) and vaccination strategies are not possible. Therefore, the eradication of a molluscs disease is a very difficult task mainly because there is not enough knowledge on the life cycle of the parasites. There have been same attempts to eradicate molluscs pathogens with no success. In the 1980's bonamiosis was introduced to the Dutch flat oyster stocks with devasting consequences. Attempts to eliminate the disease by cleaning sites and restocking with disease free animals failed. Van Banning (1990, 1991) demonstrated that, when an oyster bed was cleaned and fallowed, the naïve oysters reintroduced into the areas, quickly developed the infection, suggesting that a reservoir of infection had persisted in the areas. The pathogen, *B. ostreae*, was thought to be eradicated from the Limfjord in Denmark due to the extreme climatic conditions like ice winters over several consecutive years, either by direct elimination of the parasite or by elimination of the host, previously imported French oysters, thus preventing the spread of *Bonamia* parasites to the naïve flat oyster population in the Limfjord (Madsen et al., 2013). Nevertheless, *B. ostreae* was detected for the first time in the naïve European flat oysters from Denmark in autumn 2014 although no increased mortalities were found in connection with this finding (Madsen, 2015).

Because of that, health management policies in areas where disease outbreaks occur are focused in the importance to reduce the incidence or the severity of a disease (Grizel et al., 1986) using different strategies; changes in culture management procedures (Korringa, 1950; Lauckner, 1983; Andrews and Ray, 1988; Montes et al., 2003), increasing the tolerance of the individuals to disease through stimulating the immune system (Macey and Coyne, 2005; Xue et al., 2008; Van Hai et al., 2009), producing tolerant strains by genetic handling (Ford and Haskin, 1987; Martin et al., 1993; Gaffney and Bushek, 1996; Hand et al., 1998; Nell et al., 2000; Culloty et al., 2004; Nell and Perkins, 2006; Samain et al., 2007; Villalba et al., 2007; Dove et al., 2013a,b; Lynch et al., 2014) or replacing susceptible species with resistant ones (Grizel and Héral, 1991; Man et al., 1991). However, the introduction of foreign species is a risky task that could introduce new pathogens, predators, or competitors that may perturb the equilibrium of the ecosystem and affect adjacent areas or countries (Carriker, 1992; Shatkin et al., 1997).

Taking into account all these considerations, the effectivity of health management measures to avoid economic losses and to avoid the spread of the disease to a disease free areas are directly related with the early diagnosis of the diseases using sensitive and rapid diagnostic techniques, the collection of diagnosis data, and the establishment of diagnostic laboratories using standardized procedures with samples collected according to defined rules.

Lately, there has also been tentative studies on predictive models using epidemiological principles to design surveillance plans to evaluate the most probable time of the year when a pathogen can be detected (Lupo et al., 2014; Pande et al., 2015). Besides, the development of molecular biology tools applied to diseases processes in marine bivalve molluscs is giving us large information mainly in the study of immunity and host–pathogen interactions in molluscan bivalves that it is being applied in the development of disease resistant strains and the development of new diagnostic tools (Gomez-Chiarri et al., 2015).

## From histopathology to molecular tools

The methods of diagnosis of the diseases of the aquatic animals has evolved rapidly. Historically, most of the descriptions of molluscs pathogens are based on structural and ultra-structural studies. Traditionally, the diagnosis of molluscs diseases has primarily been achieved using histological methods and transmission electron microscopy (Azevedo et al., 1990; Anderson et al., 1994; Hervio et al., 1996; Longshaw et al., 2001; Howard et al., 2004). Histology provides a large amount of information not only of the general health of a shellfish but also the detection of a wide range of pathogens specially protozoan parasites associated to mortalities or alterations in the quality of cultured populations, or lesions associated to the interaction of the pathogen with the molluscs immune system. However, histopathology is time consuming and requires professional training and therefore many pathogens are difficult to detect by this method if there are low numbers of parasites present within infected tissues. It can also be difficult to definitively diagnose infections based exclusively on parasite species morphology criteria when there are similar morphological characteristics between species (Hine et al., 2001; Abollo et al., 2008). This is especially relevant when dealing with microcell parasites. Abollo et al. (2008) described slight differences between *B. ostreae* and *B. exitiosa* detected in the European flat oyster, *Ostrea edulis*, when observed at the microscope. *B. ostreae* shows a peripheral nucleus and scant cytoplasm while *B. exitiosa* shows a central nucleus, sometimes subcentral but rarely peripheral, and the cytoplasm larger than the one observed in *B. ostreae*.

To overcome these problems, from the 90s to nowadays, there has been a rapid increase of the use of molecular techniques for diagnosing bivalve infectious agents. Since 1997–2011 there have been published more than 2000 research papers trying to detect, identify or quantify parasites or diseases organisms carried out by ecto-parasites using DNA based tools (Hunt, 2011). These techniques are in comparison with many traditional methods, more rapid, sensitive, and profitable, and they do not demand the presence of too specialized personnel. DNA is a useful molecule to target for diagnostic procedures because its sequence does not usually vary with the life stage or developmental phase of the pathogen or with the host or tissue location (Mialhe et al., 1995).

The amplification of DNA fragments of a pathogen and subsequent sequence analysis can help us to confirm infections in different hosts (Abollo et al., 2008; Grijalva-Chon et al., 2015). This is of particular interest considering that, apparently, pathogens such as *M. refringens* have been detected in different molluscs species not showing a tight host specificity (Berthe et al., 1999, 2004). The European Directive 2006/88/EC has stablished a list of susceptible species for each of the notifiable pathogens, and based on molecular data this list should be actualized. That is the case of the protozoan parasite *B. exitiosa* listed in the Directive as an exotic disease. The pathogen was known to infect *Ostrea chilensis* in New Zealand (Hine et al., 2001; Berthe and Hine, 2004) and *Ostrea angasi* in Australia (Corbeil et al., 2006a) causing mass mortalities. However, molecular analyses have led to detection of *B. exitiosa* naturally infecting the European flat oyster *O. edulis* in Galician (NW Spain) coastal waters (Abollo et al., 2008) for the first time. Since this detection, the parasite has also been reported from different European countries infecting *O. edulis* (Narcisi et al., 2010; Arzul et al., 2012; Carrasco et al., 2012a; Longshaw et al., 2013; Batista et al., 2016).

Nevertheless, legislation is not flexible and is slow reacting to new pathological situations and because of that, at present time, *B. exitiosa* is still considered as an exotic disease to the EU.

It is also important to emphasize the potential of these new detection tools for elucidating the life-cycle of the parasites, crucial for the control and prevention of the diseases. These techniques offer the advantages of high sensitivity and high specificity, and possible rapid screening of aquatic organisms for the presence of the pathogen even in water samples. In that way, PCR was first used to screen every species sampled in the claire ponds for the presence of the parasite *M. refringens*. The copepod *Paracartia* (*Acartia) grani* as potential hosts (Audemard et al., 2002). Carrasco et al. (2007) also detected the presence of the pathogen in six different zooplankton taxa including copepods and larval stages of decapod crustaceans, although their role as intermediate host have not already being investigated. More recently, Boyer et al. (2013) and Arzul et al. (2014) confirmed by *in situ* hybridization the presence of *M. refringens* in the copepod *Paracartia grani* and *Paracartia latisetosa* respectively. Although it is not a notifiable disease for the EU, based also on molecular tools, the parasite *Marteilia sydneyi*, the aetiological agent of QC disease in the Sydney rock oyster (*Saccostrea glomerata*), was also identified in the epithelium of the intestine of two specimens of the polychaete worm, *Nephtys australiensis* (Adlard and Nolan, 2015), its detection was also confirmed by *in situ* hybridization. These new findings have contributed to a better knowledge of the disease.

Molecular tools can also circumvent problems inherent in study of organisms for which no *in vitro* culture medium is available as the case of molluscs viruses. The lack of established cell lines from mollucs difficults their isolation and subsequent study.

## The other side of using molecular tools as diagnostic techniques

However, as review by Burreson (2008), the detection of the pathogen's DNA by the polymerase chain reaction (PCR) indicates the presence of the infectious agent but it does not confirm that the pathogen is in a viable form causing an actual infection and no information is given about the tissue localization of the pathogen. Because of that, it is important to confirm the results by histology or *in situ*- hybridization (ISH) to verify that the pathogen really affects the mollusc. ISH, first developed by Joseph G. Gall (Gall and Pardue, 1969), is the most adequate technique for this purpose because it offers the advantages of the histology and the specificity of the molecular tools, but it is time consuming and its use is not recommended as a technique in surveillance programs.

In addition, the routine use of DNA based diagnostic techniques is hampered by a number of problems, which may result in false positive or false negative results. The design of these diagnostic techniques for molluscs diseases requires the search of molecular markers of certain specie-specific regions of the genome of the targeted pathogen (López-Flores et al., 2007a). Not all regions of the DNA are equally useful as targets for probes and/or PCR primers. rRNA genes are useful targets for diagnostic tests because there are many copies in the genome, which can help to ensure good sensitivity, and they offer a mosaic of conserved and variable regions which allow analyses at various levels of resolution. As a general rule, regions of the small-subunit and large–subunit ribosomal RNA genes (18 and 28S) are used to design probes specific as a genus level while regions in the intergenic spacers and internal transcribed spacers (IGS and ITS1-2) are most commonly used to design probes at an specie-specific level. These intergenic regions do not have a codificating function and they show a greater divergence even among close related species (López-Flores et al., 2007b). Efforts are being made to obtain sequences of the same regions from related organisms to optimize the chance of developing probes and PCR primers with the desired specificity. The development of species specific molecular diagnostic tools will be facilitated as sequences for more genes and pathogens become known. However, in order to minimize the possibility of species-specific molecular diagnostics failing to detect a particular strain of a pathogen, as many strains as possible from a wide geographic range should be sequenced. This is an important issue specially when the target strain of pathogen is pathogenic, while close relatives are harmless.

On the other hand, false-negatives results can be obtained from sampling error due to small abundance of pathogen DNA especially when pathogen is localized in an specific organ of the host, as it is the case of *M. refringens* and the tissue is subsampled, or due to the presence of inhibitors that may be present in the sample and could affect the activity of the DNA polymerase.

Specificity is designed as the ability of an assay to amplify DNA only from the target agent. Sensitivity (or limit of detection) is defined as the smallest quantity of DNA that can be systematically detected. The highly sensitivity that makes PCR a very powerful technique for amplification of DNA can also be seen as a negative aspect because of inadvertent contamination by nucleic acids. Even very low levels of contamination can result in false-positive results. Consequently, qualification of the specificity and quantification of the sensitivity of a molecular assay is required and as the assay only detect nucleic acid not assuming a real infection, it needs to be validated against other techniques, such as histology or *in situ* hybridization, so that its reliability can be determined (Kleeman et al., 2002; Carnegie et al., 2003; Burreson and Ford, 2004; Burreson, 2008). This is specially important in the detection of the pathogen in new host species or in a new geographic location.

## Available molecular tools for non exotic molluscs diseases listed by EU

### Detection of *Marteilia refringens*

*Marteilia refringens* is a protozoan parasite of the phylum Cercozoa and order Paramyxida (Cavalier-Smith and Chao, 2003; Feist et al., 2009) infecting the digestive system of several bivalve species and inducing physiological disorders and eventually death of the animal (Grizel et al., 1974; Alderman, 1979). In the case of paramyxeans, molecular studies have focused on the small-subunit ribosomal RNA gene (Le Roux et al., 1999; Berthe et al., 2000; Kleeman and Adlard, 2000; Itoh et al., 2003). However, closely related organisms, such as *M. refringens* and *M. maurini*, presently considered to be the same specie, (Le Roux et al., 2001) or *M. refringens* and *M. sydneyi* (Kleeman et al., 2002), have a high degree of sequence similarity, making the development of specific probes and primers more difficult. There has also been described PCRs focused on the ITS-1 region (Le Roux et al., 2001) and the IGS region of the parasite (López-Flores et al., 2004) although they are neither specie-specific as the newly described pathogen, *Marteilia cochillia*, is also amplified (Carrasco et al., 2012b).

At present time there is no a *M. refingens* specie-specific PCR available and an restriction analyse assay (RFLP) based on the PCR described by López-Flores et al. (2004) is needed to discriminate between *Martelia cochillia* and *Martelia refringens* (Carrasco et al., 2012b).

### Detection of *Bonamia ostreae* and *Bonamia exitiosa*

*Bonamia ostreae* and *B. exitiosa* are *Haplosporidian* protozoan parasites (Carnegie and Cochennec-Laureau, 2004) infecting haemocytes of several oyster species and inducing physiological disorders and eventually death of the animal (Grizel, 1985; Dinamani et al., 1987; Cranfield et al., 2005).

In the case of *Bonamia* species several PCR assays have been developed targeting the 18S rDNA gene, (Carnegie et al., 2000; Cochennec et al., 2000; Abollo et al., 2008), or TaqMan® assays (Corbeil et al., 2006b; Marty et al., 2006). However, none of these methods are able to differentiate between *Bonamia* species. For this reason, differentiation between species has been achieved using PCR-restriction fragment length polymorphism (PCR-RFLP) assays (Hine et al., 2001; Cochennec-Laureau et al., 2003; Abollo et al., 2008), sequencing the products obtained by PCR assays or by means of light microscopic techniques, thus causing the diagnostic process to be more expensive and slower. Recently species- specific quantitative real time PCR assays for *B. ostreae* have finally been developed targeting a region of the actin-1 gene (Robert et al., 2009) and the 18S-ITS 1 rDNA region (Ramilo et al., 2013). Other approaches has also been reported to face the problem of distinguishing among species based on DNA sensors. DNA biosensors are analytical devices resulting from the integration between DNA sequence-specific probe with a signal transducer. Narcisi et al. (2011) developed six single strand DNA probes selected within different regions of *B. ostreae* and *B. exitiosa* with the aim to discriminate between both species. A post PCR nucleic acid work by comparing experimental data, from electrochemical genosensors, and bioinformatics data, derived from the simulation of the secondary structure folding and prediction of hybridization reaction, showed correspondence indicating the possibility to use the prediction method for ssDNA probes selection in the genosensors development.

Quantitative real time PCR is used to amplify and simultaneously detect and quantify a targeted DNA molecule following the cycling principle of basic PCR. It does not require post-PCR sample handling to visualize the result in an agarose gel, preventing potential PCR product carry-over contamination resulting much faster and higher throughput assays. The amplified DNA is detected as the reaction progresses in “real time” in contrast with the standard PCR where the product of the reaction is detected at the end. The data thus generated is analyzed by computer software to detect and quantify the targeted DNA in samples to determine the presence and abundance of a particular pathogen. Different quantitative real time PCRs have already been described to detect molluscs pathogens (Corbeil et al., 2006b; Marty et al., 2006; Robert et al., 2009; Martenot et al., 2010; Umeda and Yoshinaga, 2012; Carrasco et al., 2013; Ramilo et al., 2013). There have also been attempts to develop multiplex PCRs to detect the presence of different molluscs pathogens in the same reaction in order to facilitate their diagnosis (Penna et al., 2001; Xie et al., 2010, 2013; Umeda and Yoshinaga, 2012). Recently, Ramilo et al. (2013) has developed a specie-specific multiplex PCR for the diagnosis of *B. ostreae* and *B. exitiosa* simultaneously, using a cocktail of 3 primers. When there is coinfection of both species, there is competition for the forward primer between them that targets the same region of the 18S rDNA gene of both species, and for other reaction components frequently leading to detectable amplification of DNA of just one of the parasites, suggesting that the multiplex PCR assay should be improved.

## Conclusion, open questions and way forward

Valid laboratory results are essential for diagnosis, surveillance, and trade. Although molecular tools are now moving from a developmental phase in specialized laboratories for research purposes to routine application, they still need formal validation. These diagnostic techniques are not standardized and differences exist in the quality of reagents, sample preparation, in controls, as well as in the interpretation of the results. The use of a “standardized” diagnostic tool for routine analysis should allow the implementation of a calibrated and controlled process in laboratories. It is recognized that for such purpose, studies conducted in parallel with the same isolates in several laboratories would be necessary. Balseiro et al. (2006) and Flannery et al. (2014), carried out respectively comparative studies of the different diagnostic techniques between different laboratories to detect *B. ostreae*, standing out the importance of the molecular tools. However, PCR used in those studies was not *B. ostreae* specie-specific.

The European Union Reference Laboratory (EURL) for mollusc diseases (IFREMER) is the reference institution in charge of the harmonization of the molluscs diagnostic techniques for the EU listed pathogens. To carry out this task, it has elaborated standard operating procedures in which protocols specifying the procedures from the tissue sample to the reactives used in pathogen diagnosis are summarized and organizes regularly interlaboratory comparison tests among all interested national reference laboratories in EU. In 2013 the EURL organized a qPCR interlaboratory comparison test for the detection of *Bonamia* sp., and the characterization of the species *B. ostreae* and *B. exitiosa*. Optimal results were obtained and at present time the multiplex qPCR designed by Ramilo et al. (2013) for the diagnosis of *B. ostreae* and *B. exitiosa* simultaneously is now recommended in the Commission Implementing Decision (EU) 2015/1554^3^ as regard requirements of diagnostic tools. However, comparative studies among different laboratories of the different diagnostic techniques are still missing in order to validate these newly described molecular tools.

The development of the molecular tools has significantly improved the diagnosis of molluscs diseases due to their high specificity and sensitivity, higher reproducibility of the results and faster detection of the pathogen. In Table 2 it is summarized the recommended diagnostic methods for the diagnosis of molluscs diseases recommended by the OIE of the EU listed diseases by Council Directive 2006/88/EC^4^ taking into account the final purpouse of the diagnosis. Methods are classified depending if they are going to be used in a targeted surveillance program, as a presumptive diagnosis or as a confirmatory diagnosis. Methods used in targeted surveillance programs should not be time consuming and in most of the cases, the use of molecular tools is recommended.

**Table 2:** **Available diagnostic techniques recommended by O.I.E. (2016) for molluscs pathogens listed by EU**.

	**Targeted surveillance**		**Presumptive diagnosis**		**Confirmatory diagnosis**	
	**Recommended method**	**Standard method**	**Recommended method**	**Standard method**	**Recommended method**	**Standard method**
*B. ostreae*	PCR (L, P, J, A) qPCR (L, P, J, A) Histopathology (J, A) Tissue imprints (J,A)		Tissue imprints PCR qPCR	Histopathology	TEM sequencing	PCR-RFLP ISH
*B. exitiosa*	PCR (L, P, J, A) qPCR (L, P, J, A) Histopathology (J, A) Tissue imprints (J,A)		Tissue imprints PCR qPCR	Histopathology		
*M. refringens*	PCR (L, P, J, A) Histopathology (J, A)	Tissue imprints (J, A)	PCR tissue imprints	Histopathology	PCR sequencing	ISH TEM
*M. mackini*	Histopathology (A)	PCR (J, A)		PCR Histopathology	ISH sequencing	Histopathology TEM PCR
*Perkinsus marinus*	PCR (L) RFTM (J, A)	Histopahology (P, J, A) PCR (J, A) ISH (J, A)	PCR	ISH Histopathology RFTM	ISH	PCR Sequencing
Herpes virus μvar	PCR (L, J, A) qPCR (L, J, A)		PCR (L, J, A) qPCR (L, J, A)		Sequencing	PCR qPCR ISH TEM

*A, Adults; P, post-larvae; L, Larvae; J, Juveniles*.

Likewise, in the last few years, there have been greatly advances in the development of molecular tools with the use of new- generation sequencing technologies (NGS) and their ability to produce large volumes of data. The use of these technologies has drastically increased the number of molluscs genomic sequences in the databases.

Genomic research is basic to solve specific problems in bivalve aquaculture, including disease control. They can identify RNAs or proteins expressed in response to stress or pathogens and compensate the usual absence of clinical manifestations. All this new information will facilitate the study of bivalves and increase the success of molluscan aquaculture, facilitating the monitoring of the production, activation state of pathogens or immune status of the animals. Up to now, these methodologies have been applied to select key genes linked to resistence that could be used as markers for selection (Romero et al., 2012; Gomez-Chiarri et al., 2015), identify genes implicated in the immune response (Philipp et al., 2012; Moreira et al., 2012, 2014; Zhang et al., 2014; Gomez-Chiarri et al., 2015), or investigate biological processes (Clark et al., 2010; Joubert et al., 2010). Genomic advances happen in an exponential way. In the next incoming years the amount of available information will allow reserchers huge progresses regarding molecular biology of bivalve molluscs and pathogens.

## Author contributions

RA conducts the literature review and writing up of the paper and AF contributes to the revision of the paper.

### Conflict of interest statement

The authors declare that the research was conducted in the absence of any commercial or financial relationships that could be construed as a potential conflict of interest.

## Footnotes

^1^World Organisation for Animal Health. OIE. Manual of diagnostic test for aquatic animals. Chapter1.1.2. access online.

^2^Commission regulation (EU) No. 175/2010 of 2 March 2010 implementing Council Directive 2006/88/EC as regards measures to control increased mortality in oysters of the species Crassostrea gigas in connection with the detection of Ostreid herpesvirus 1 μvar (OsHV-1 μvar).

^3^Commission Implementing decision (EU) 2015/1554 of 11 September 2015, laying down rules for the application of Directive 2006/88/EC as regards requirements for surveillance and diagnostic methods.

^4^Council Directive of 24 October 2006 on animal health requirements for aquaculture animals and products thereof and on the prevention and control of certain diseases in aquatic animals.
